# Parental Imbalances Involving Chromosomes 15q and 22q May Predispose to the Formation of *De Novo* Pathogenic Microdeletions and Microduplications in the Offspring

**DOI:** 10.1371/journal.pone.0057910

**Published:** 2013-03-06

**Authors:** Valeria Capra, Samantha Mascelli, Maria Luisa Garrè, Paolo Nozza, Carlotta Vaccari, Lara Bricco, Frédérique Sloan-Béna, Stefania Gimelli, Cristina Cuoco, Giorgio Gimelli, Elisa Tassano

**Affiliations:** 1 Unità Operativa di Neurochirurgia, Istituto Giannina Gaslini,Genova, Italy; 2 Modulo Dipartimentale di Neuro-Oncologia, Istituto Giannina Gaslini, Genova, Italy; 3 Unità Operativa di Anatomia Patologica, Istituto Giannina Gaslini, Genova, Italy; 4 Molecular Genetics and Cytogenetics Unit, Istituto Giannina Gaslini, Genova, Italy; 5 Service of Genetic Medicine, University Hospitals of Geneva, Geneva, Switzerland; 6 Laboratorio di Citogenetica, Istituto Giannina Gaslini, Genova, Italy; IGBMC/ICS, France

## Abstract

Microarray-based comparative genomic hybridization (array-CGH) led to the discovery of genetic abnormalities among patients with complex phenotype and normal karyotype. Also several apparently normal individuals have been found to be carriers of cryptic imbalances, hence the importance to perform parental investigations after the identification of a deletion/duplication in a proband. Here, we report the molecular cytogenetic characterization of two individuals in which the microdeletions/duplications present in their parents could have predisposed and facilitated the formation of *de novo* pathogenic different copy number variations (CNVs). In family 1, a 4-year-old girl had a *de novo* pathogenic 10.5 Mb duplication at 15q21.2q22.2, while her mother showed a 2.262 Mb deletion at 15q13.2q13.3; in family 2, a 9-year-old boy had a *de novo* 1.417 Mb deletion at 22q11.21 and a second paternal deletion of 247 Kb at 22q11.23 on the same chromosome 22. Chromosome 22 at band q11.2 and chromosome 15 at band q11q13 are considered unstable regions. We could hypothesize that 15q13.2q13.3 and 22q11.21 deletions in the two respective parents might have increased the risk of rearrangements in their children. This study highlights the difficulty to make genetic counseling and predict the phenotypic consequences in these situations.

## Introduction

Among patients presenting with a complex phenotype characterized by multiple congenital anomalies and intellectual disability (ID)/developmental delay (DD) and with a normal karyotype, an average of 11–17% of significant genomic abnormalities were reported using array-CGH screening. Moreover, cryptic imbalances have also been identified in normal individuals [1]–[3].

The imbalances associated with human diseases, termed “genomic disorders”, are usually mediated by flanking blocks of duplicated sequences which predispose specific chromosomal regions to highly frequent rearrangements via non-allelic homologous recombination (NAHR), leading to deletions, duplications, or inversions of the intervening sequences [4]–[5].

Chromosome 22q11.2 region contains eight different chromosome 22-specific low-copy repeats, designated LCR22s (LCR22-A-LCR22-H), which are known to mediate recurrent microdeletions and microduplications by NAHR.

The long arm of chromosome 22 at band q11.2 can be classified as one of the most unstable regions of the genome containing unusual DNA configuration. Similarly, NAHR between segmental duplications in proximal chromosome 15q breakpoint (BP) regions can lead to microdeletions and microduplications. Recently, several patients with a recurrent 15q13.3 microdeletion syndrome (MIM 612001) has been reported. There is a shared ∼2.0 Mb region between segmental duplications at BP4 and BP5 containing six known genes and the expressivity of the condition appears quite variable.

Similar unstable regions are present on several other chromosomes (e.g. chromosome 8 at band p23.1p23.2, chromosome 15 at bands q11q13, q24q26, and chromosome 17 at band p11.2) [4].

In this study, we report on phenotype, cytogenetic and molecular findings in two patients and discuss if the microdeletions/duplications present in their parents could have predisposed and facilitated the formation of the *de novo* pathogenic copy number variations (CNVs).

## Results

### Family 1

#### Clinical Report

The patient is a 4-year-old female born from to non consanguineous parents. Her elder sister presented mild psychomotor retardation. Her mother presented preauricular tag and a normal psychomotor development. She was born at 38^th^ weeks of pregnancy by caesarean section for intrauterine growth retardation. Exposure to drugs or infections during pregnancy was denied. At birth, weight was 2200 g (<3^rd^ centile), length 47 cm (10^th^–25th centile) and occipito-frontal circumference 32 cm (5^th^ centile). Her psychomotor development was retarded; at the age of 4 years, she attended nursery school with teacher support. The child has a vascular malformation in the left maxillary sinus, detected occasionally by facial CT and MRI after trauma, consistent with suspected capillary hemangioma. Ophthalmologic evaluation detected esophoria, astigmatism and hypermetropia. At our physical examination, at the age of 4 years, weight was 12.3 kg (3^rd^ centile), height 100 cm (50^th^ centile) and occipitofrontal circumference 44.5 cm (<3^rd^ centile). It was possible to further detect two preauricular tags and a preauricular pit.

**Figure 1:**
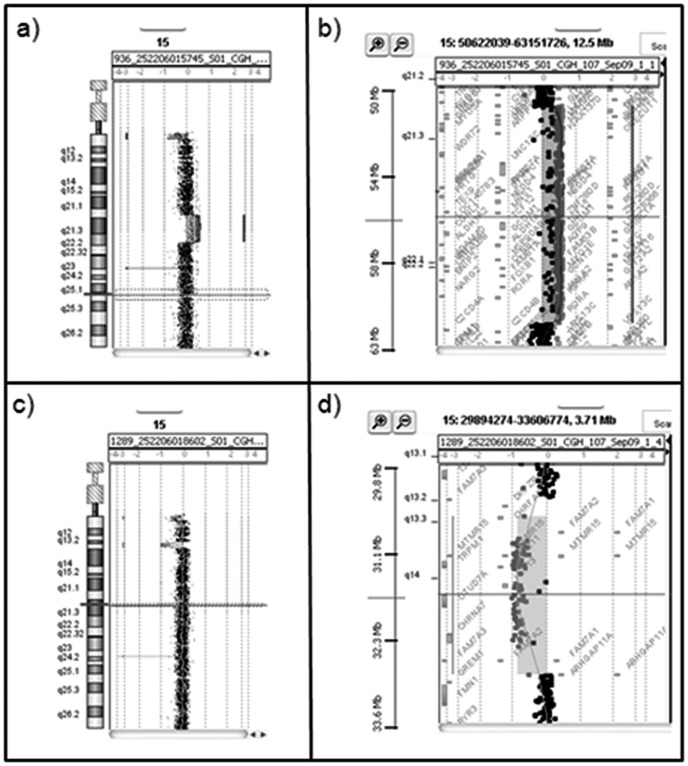
Family 1, array-CGH results. a) Array-CGH graphical overview of 15q21.2q22.2 duplication of the proposita. b) Enlargement of the duplicated region 15q21.2q22.2 spanned 10.5 Mb from oligonucleotide A_16_P03028573(51,604,508 bp) to A_16_P20273880 (62,102,756 bp). c) Array-CGH graphical overview of 15q13.2q13.3 deletion of the mother. d) Enlargement of the deleted region spanned 2.262 Mb from oligonucleotide A_18_P12164655 (30,652,489 bp) to A_14_P200747 (32,914,140 bp).

**Figure 2:**
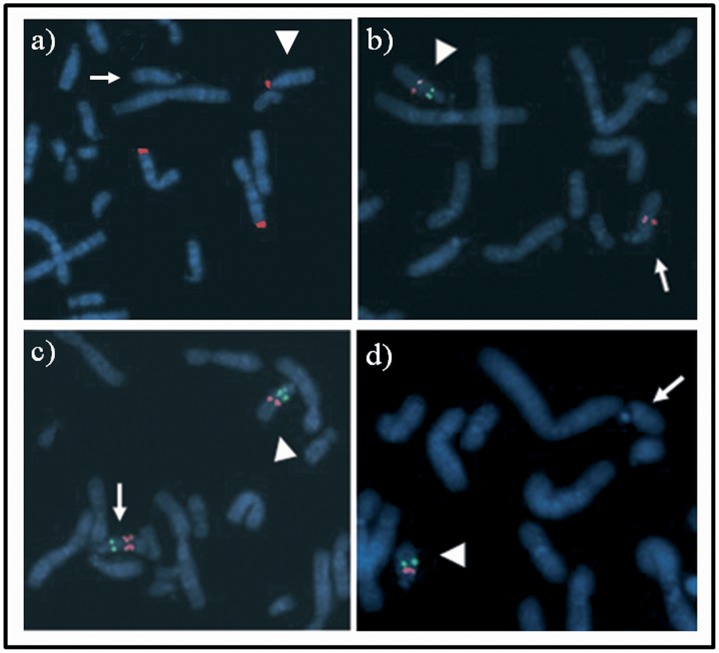
FISH results. a) FISH with specific subtelomeric 7q probe (red) confirmed the presence of the unbalanced translocation both in the patient and mother. b) FISH with oligonucleotide-based probe 15q13.3 (green signal) and BAC RP11-231A23 (red signal) on the metaphase of the mother in family 1 showing the deletion on a chromosome 15 (arrow). The arrowhead indicates the normal chromosome 15. c) FISH with oligonucleotide-based probe 15q13.3 (green signal) and BAC RP11-231A23 (red signal) on the metaphase of the proband in family 1 showing the duplication on a chromosome 15 (arrow). The arrowhead indicates the normal chromosome 15.FISH performed with the same probes on the metaphase of the mother showed normal signals only on both chromosome 15 (data not showed). d) FISH with oligonucleotide-based probe 22q11.21 (red signal) and BAC RP11-1143M16 (green signal) on the metaphase of the proband in family 2 confirming that large and short deletions are on the same chromosome 22. Arrowhead shows the normal chromosome 22 and arrow the deleted chromosome 22.

Chromosome analysis of the child showed a subtle unbalanced translocation between the long arm of a chromosome 7 and the short arm (satellites?) of a chromosome 14. FISH analysis with a specific subtelomeric 7q probe confirmed the presence of the unbalanced translocation in both patient and mother.The older sister and her father had normal results. To further characterize rearrangement extent and breakpoints, array-CGH analysis was performed, showing a duplicated chromosomal terminal segment on 7q36.3 spanning 526.9 Kb according to NCBI Build 37 (GRCh37)(Feb. 2009). The same translocation was also present in the mother. Furthermore, our patient, presented a duplication of 10.5Mb (chr15∶51,604,508–62,102,756) on chromosome 15q21.2q22.2 (Fig. 1, A–B). This duplication was not present in the mother. FISH performed with probes mapping in 15q21.2q22.2 duplicated region showed signals only on both chromosome 15, excluding possible balanced rearrangements involving other chromosomes (data not showed). Microsatellite analysis of D15S970 for the patient showed that the duplicated allele was of maternal origin. Unexpectedly, array-CGH in her mother showed a deletion of 2.262 Mb (chr15∶30,652,489–32,914,140) on chromosome 15q13.2q13.3 described as “recurrent 15q13.3 microdeletion syndrome” [6]. (Fig. 1, C–D). These data were confirmed by FISH experiments (Fig. 2, A–B).

**Figure 3:**
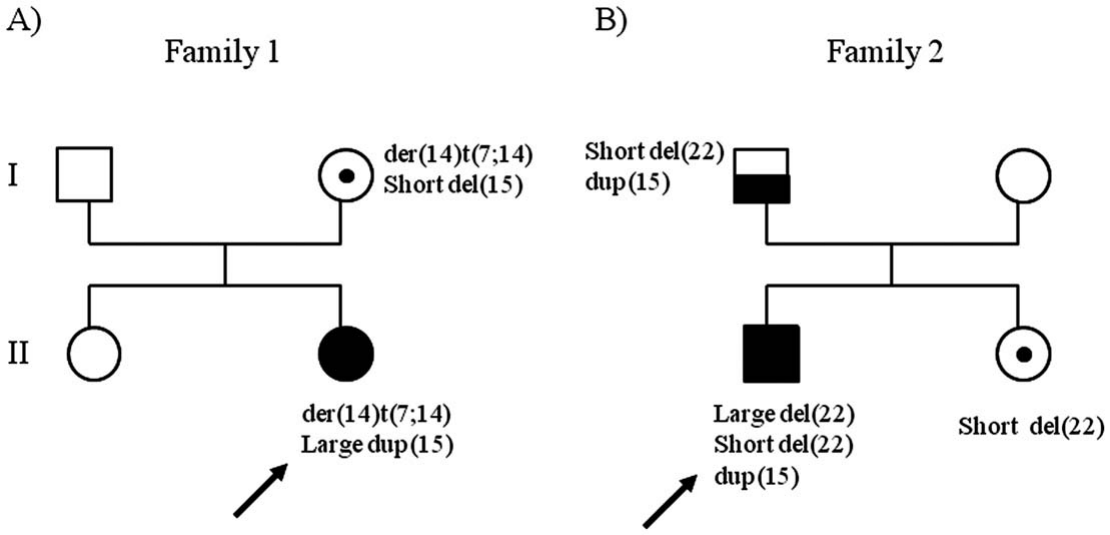
Pedigrees of families with inherited deletions/duplications. (A) Pedigree of Family 1. The proposita (II, 2) carried an unbalanced 7/14 translocation and a *de novo* large 15q21.2q22.2 (10.5 Mb) duplication. The mother (I, 2) carried the same unbalanced 7/14 translocation and a BP4–BP5 15q13.2q13.3 deletion of 2.262 Mb (short deletion). B) Pedigree of Family 2.The propositus (II, 1) had two deletions on the same chromosome 22: a short 22q11.23 (247 Kb) deletion inherited from his father and one *de novo* large deletion 22q11.21 (1.417 Mb). He also showed a duplication of 484.3 Kb at 15q26.3 inherited from his father. His normal sister (II, 2) carried only the short deletion 22q11.23. Their father (I, 1) had both the short deletion of chromosome 22 and the duplication of chromosome 15.

In summary, our patient carries an unbalanced translocation 7/14 inherited from her mother and a duplication of 10.5 Mb on chromosome 15q21.2q22.2. In addition to the unbalanced translocation, the patient’s mother carries a deletion of 2.262 Mb on chromosome 15q13.2q13.3 (Fig. 3, A).

### Family 2

#### Clinical report

The patient was a 9-year-old male referred at the age of six for orthopedic assessment because of right leg pain and gait disturbance. He was the eldest child of non-consanguineous parents. Maternal family history was unremarkable except for a second degree uncle with pes cavus. The parents were both healthy and the father reported that he was macrosomic at birth. Pregnancy was full term and delivery was uneventful. The patient’s birth weight was 4270 g (>90^th^ percentile), length 53 cm (>90^th^ percentile). The patient’s medical history revealed a mild delay in motor milestones, he started walking at 18 months. Speech acquisition was also delayed. At the age of 8 years, neurological examination confirmed motor awkwardness, psychomotor delay, and gait disorder (walking on toes and pes cavus). Cerebro-spinal MRI revealed swelling at D8-L1, hyperintense on T2-weighted images with irregular enhancement, ascribable to expansive intramedullary lesion without CNS dissemination. Bilateral dysgenesis of the lateral semicircular canal of the inner ear was also associated. Abnormalities of gyration and abnormal foliation of some sectors of both cerebellar hemispheres were also observed. The patient underwent to biopsy. Histological examination identified a pilocytic astrocytoma (WHO grade I). At physical examination, mild facial dysmorphisms was observed. The child presented low and flat forehead, very arched eyebrows, small nose with large filter, small mouth, downslanting and curled ears, short neck. Additionally, the child had small hands with flared distal phalanges, “drumstick-like”), and cafe-au-lait spots on the back. Auxological examination revealed mild obesity. The parents gave written informed consent for genetic studies.

**Figure 4:**
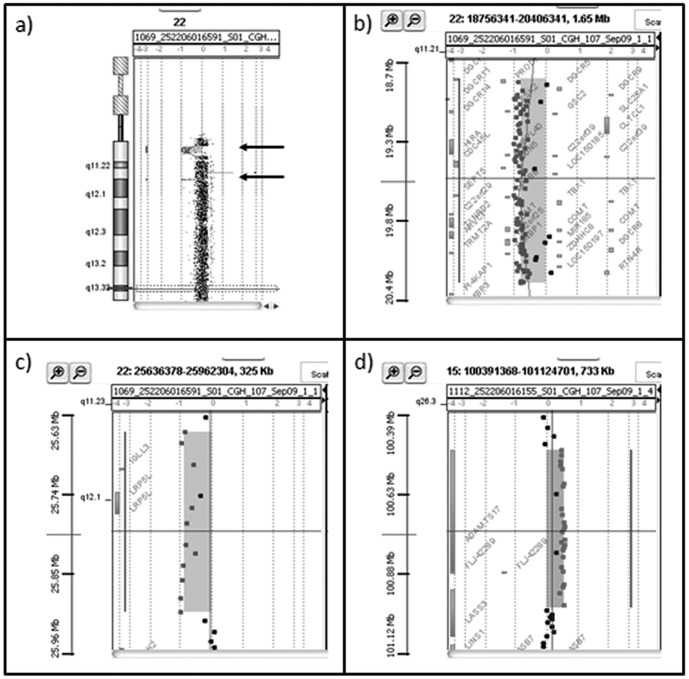
Family 2, array-CGH results. a) Array-CGH graphical overview of 22q11.21 deletion (large deletion) of the proband. b) Enlargement of the deleted region spanned 1.417 Mb from oligonucleotide A_16_P41477688 (18,894,835 bp) to A_14_P200761(20,311,763 bp). c) Enlargement of the 22q11.23 deleted region (short deletion) spanned 247 Kb from oligonucleotide A_14_P133174 (25,664,618 bp) to A_18_P13974242 (25,911,651 bp) inherited from his father. d) Enlargement of the 15q26.3 duplicated region spanned 484.3 Kb from oligonucleotide A_16_P20368426 (100,051,182 bp) to A_14_P110953 (100,996,155 bp) of the proband and inherited from his father.

Array-CGH analysis, performed on the proband, revealed the presence of two deletions of chromosome 22 and a duplication of chromosome 15. The extent of the deletion at 22q11.21 band (large deletion) was 1.417 Mb (chr22∶18,894,835-20,311,763) (Fig. 4, A–B) while the second deletion at band 22q11.23 (short deletion) (chr22∶25,664,618-25,911,651) spanned 247 Kb (Fig. 4, C). Furthermore, the patient carried a duplication spanning 484.3 Kb (chr15∶100,051,182-100,996,155) at q26.3 band of a chromosome 15 (Fig. 4, D). The short deletion of chromosome 22 and the duplication of chromosome 15 were inherited from his apparently normal father, while his phenotypically normal sister inherited only the short deletion. His mother had no abnormalities (Fig. 3, B). The large deletion of chromosome 22 was described as proximally nested 1.5 Mb deletion harboring TBX1 and is present in 7–14% of DiGeorge/Velo-cardio-facial patients (DGS/VCFS) [7]–[10]. FISH experiments confirmed the presence of the short deletion on the metaphases of the father and showed that the large and short deletions were both on the same chromosome 22 on the metaphases of the son (Fig. 2, C).

## Discussion

The application of array-CGH for the assessment of children with congenital anomalies and/or idiopathic mental retardation has facilitated the identification of new multiple microdeletion/microduplication syndromes. In a number of cases, the deleted or duplicated regions are inherited from an apparently normal parent. It is well known that apparently unaffected parents or with a mild phenotype and carriers of a deletion or a duplication can transmit the imbalances to the offspring who can be more severely affected. Several possibilities may account for phenotypic variability including variation in genetic background, epigenetic phenomena, gene expression, and/or regulatory variation among genes in the rearranged regions [11]–[13]. In particular, the genomic copy number changes seen in 1q21.1, 1q41q42, 3q29, 15q11.2, 15q13.2q13.3, 16p11.2, 16p13.11, and 22q11.2 have been considered to have incomplete penetrance and variable expressivity [3].

In this paper, we describe two families in which the index cases had an apparently *de novo* imbalance involving either chromosome 15q or 22q. In both cases, one of the parents carried a microdeletion/duplication on the same chromosome.

In family 1, a 4-year-old girl had a *de novo* 10.5 Mb duplication in 15q21.2q22.2, while her mother showed a 2.262 Mb deletion in 15q13.2q13.3. Chromosome 15 is one of the seven human chromosomes with a high rate of segmental duplication [14]. LCRs in chromosome 15 are largely clustered in proximal and distal 15q [15]. The majority of the microdeletion/microduplication cases were reported in the region of Prader-Willi/Angelmann syndrome mapped at 15q11.2-q13 bands. In this region, there are two clusters of LCRs named BP1 and BP2 and one more distal single common breakpoint (BP3). Each breakpoint corresponds to a complex set of segmental duplications.

Recently, others recurrent microdeletion syndromes (15q13.3 and 15q24), all mapping to large blocks of segmental duplications, were reported in other regions of chromosome 15 [16], [6]. The most important features among the individuals with microdeletion 15q13.3 were mild to moderate mental retardation and epilepsy and/or abnormal EEG findings, but a number of phenotypically normal parents were also reported [6], [17]. In our case, the 15q13.2q13.3 deletion in the mother is flanked by more distal LCRs termed BP4-BP5, while no obvious segmental duplications have been detected (UCSC, Segmental Duplication track setting and Self Chain) at the breakpoint of 15q21.2q22.2 duplication in the proband.

The propositus of family 2, a 9-year-old boy, had a *de novo* 1.417 Mb deletion in 22q11.21 and a second paternal deletion of 247 Kb in 22q11.23. The band 22q11.2 contains one of the most rearrangement-prone regions in the human genome, where the breakpoints of a number of constitutional deletions, duplications, and translocations are localized. Rearrangements are associated with genetic disorders including DGS/VCFS 22q11.2 deletion syndrome, Cat Eye inv dup(22)(q11) duplication syndrome, and supernumerary der(22) t(11;22) syndrome [18]. The genomic structure of 22q11.2 region has a causative role in the origin of rearrangements, in fact the breakpoints are frequently localized next to LCRs, known as LCR22s, sharing modules with 97–98% sequence identity. Because of their high identity, LCR22s have been suggested to mediate deletions/duplications through NAHR events between the repeats. Chromosome 22 deletions of the DGS/VCFS region typically span a 3 Mb region in 22q11 and are flanked by the largest LCR-A and LCR-D. In a number of cases, deletions of 1.5–2 Mb flanked by LCR-A and LCR-B or LCR-A and LCR-C were reported [9], [19]. Our large deletion is flanked by LCR-A and LCR-B including *TBX1* gene and could be classified as the common smaller 22q11.2 deletion causing DGS/VCFS. Interestingly, the short deletion inherited from the patient’s father is very close to the distal end of LCR22-8 and contains numerous segmental duplications. A NAHR mechanism is likely to be involved to create the rearrangements observed in this family, as the breakpoints of 22q11.23 and 22q11.21 deletions are found to map within LCRs. The misalignment of segmental duplications and an unequal crossing over between homologous chromosomes (interchromosomal) or within a single or sister chromatid (intrachromosomal), through the formation of a deletion loop within the normal chromosome, could have mediated the generation of the large deletion in chromosome 22 in family 2. To our knowledge, this is the second report of parental imbalances influencing the rearrangement in the sibship. Recently, Tabet et al. [20] reported two monozygotic twins carrying a *de novo* 16p11.2p12.2 duplication (8.95 Mb). Their eldest brother had a smaller 16p11.2 microdeletion (847 Kb) inherited from his father. The authors concluded that the 16p11.2 deletion in the father predisposed to the duplication in his twin children.

In conclusion, we hypothesize that the identified 15q13.2q13.3 and 22q11.21 deletions in the two respective parents might have increased risk of rearrangement in their children making genetic counseling and prediction of the phenotypic consequences particularly challenging.

## Materials and Methods

### Cytogenetics and Molecular Analysis

In all patients, standard GTG banding was performed at a resolution of 400–550 bands on metaphase chromosome preparations from peripheral blood lymphocyte cultures.

Array-CGH was performed on patients and their parents using the Human Genome CGH Microarray Kit G3 180 (Agilent Technologies, Palo Alto, USA) with 13 KB overall median probe spacing. Labeling and hybridization were performed following the protocols provided by the manufacturer. Graphical overview was obtained using the CGH analytics software Genomic Workbench 6.5 - Lite Edition (Agilent). To confirm the results of array-CGH, fluorescence *in situ* hybridization (FISH) experiments were performed on metaphase chromosomes of mother and daughter of family 1 with the following probes: subtelomeric Tel7qter probe (TelVysion 7q SpectrumOrange; Vysis/Abbott, Inc., Downers Grove, IL), oligonucleotide-based probe 15q13.3 (chr15∶31,775,207-31,899,230) (SureFISH G100005G, Agilent Technologies), BAC probe RP11-231A23 (chr15∶58,181,400- 58,324,747 )(Bluegnome, Cambridge, UK) mapping at 15q22.2. The oligonucleotide-based probe 22q11.21 (chr22∶19,723,507-19,791,767) (SureFISH G100014R, Agilent Technologies) and BAC probe RP11-1143M16 mapping at 22q11.23 (chr22∶25,725,620-25,879,526) (Invitrogen, Life Technologies Co.) were used on metaphase chromosomes of father and son of family 2.

Microsatellite analysis used fluorescent-tagged PCR amplified products analysed using an ABI3100 capillary genetic analyser and Genescan and Genotyper softwares (Applied Biosystems). Parental origin of the duplicated chromosome 15 in family 1 was determined using microsatellite markers D15S1049, D15S982 and D15S970, which lie within the duplicated region.
